# Effect of total parathyroidectomy in patients with secondary hyperparathyroidism: a retrospective study

**DOI:** 10.1007/s11255-022-03401-3

**Published:** 2022-11-04

**Authors:** Xixiang Gong, Yi An Wang, Chunqi Li, Xue Liao, Shihua Li, Liping Yang, Xuelian Jiang, Yang Sun, Jianqing Xu, Zongwu Tong, Yongxin Lu

**Affiliations:** 1 Department of Otolaryngology-Head and Neck Surgery, People’s Hospital of Yuxi City, The Sixth Affiliated Hospital of Kunming Medical University, Yuxi, 653100 Yunnan China; 2 Department of Nephrology, People’s Hospital of Yuxi City, The Sixth Affiliated Hospital of Kunming Medical University, Yuxi, 653100 Yunnan China

**Keywords:** Parathyroidectomy, Secondary hyperparathyroidism, End-stage renal disease, Outcomes

## Abstract

**Purpose:**

To investigate the therapeutic efficacy, feasibility, and safety of total parathyroidectomy (tPTX) in the treatment of secondary hyperparathyroidism (SHPT).

**Methods:**

The clinical data of 34 SHPT patients admitted to the Department of Nephrology, Yuxi People’s Hospital, from January 2018 to January 2021 who had received tPTX, were retrospectively analyzed. The indications for tPTX were severe SHPT that did not respond to medical treatment and was ineligible for kidney transplantation. tPTX without autotransplantation was adopted to compare the level of symptom relief and changes in serum intact parathyroid hormone (iPTH), blood calcium, and blood phosphorus pre- and postoperatively.

**Results:**

In 34 patients, 142 parathyroid glands were removed, including 21 ectopic parathyroid glands (14.78%). Six patients (17.64%, 6/34) had supernumerary parathyroid glands. At 6 h postoperatively, arthralgia and bone pain were significantly reduced to almost zero in 94.12% (32/34) of patients. At 24 h postoperatively, relief of bone pain and improvement of limb movement were observed in 100% (34/34) of patients, and pruritus almost disappeared in 86.36% (19/22) of patients. There were significant differences in iPTH (*χ*2 = 134.93, *P* < 0.05), calcium (*χ*2 = 23.02, *P* < 0.05), and phosphorus (*χ*2 = 102.11, *P* < 0.05) levels preoperatively and 40 min, 24 h, 1 week, half a year, and last available (> 1 year) postoperatively.

The patients were followed up for 15–47 months (median 33 months). Hypoparathyroidism was observed in three patients, who underwent neck dissection or partial thymotomy concurrently for different reasons. No intractable hypocalcemia or adynamic bone disease occurred during the follow-up period.

**Conclusion:**

In SHPT patients who were ineligible for renal transplantation, tPTX was effective, safe, and reliable, with a low recurrence rate. However, when tPTX was performed alone without autologous transplantation, bilateral neck exploration was sufficient, and central neck dissection and thymic resection were inadvisable.

## Introduction

Secondary hyperparathyroidism (SHPT) is an inevitable complication in patients with end-stage renal disease on maintenance dialysis [1]. SHPT patients experience metabolic bone diseases, neurological damage, calciphylaxis, and cardiovascular system damage due to overproduction of the parathyroid hormone (PTH), which seriously affects patients’ quality of life and survival. Although a proportion of patients were able to control SHPT through blood phosphorus control and drug treatment, such as that with active vitamin D and calcium-mimetic agents (i.e., cinacalcet), a significant proportion of patients develop resistance to medications, hindering disease control with conventional therapies as the disease progresses [2]. Parathyroidectomy (PTX) is the only method for treating patients with refractory SHPT by removing the diseased parathyroid gland, which rapidly and effectively reduces PTH secretion, thereby improving the metabolism-related complications of SHPT, improving the quality of life of patients, and even reducing mortality [3].

Since it was first performed by Mandel in 1924 [4], parathyroid surgery has developed over nearly a century. Today, parathyroidectomy (PTX) is a well-established surgical technique. Despite the differing indications for PTX in different countries, the role of PTX in reducing mortality, improving prognosis [5], and reducing the financial burden [6] of SHPT patients has generally been well recognized. However, the optimal surgical procedure for the treatment of SHPT remains unclear. The main surgical options for SHPT treatment include subtotal PTX (sPTX), total PTX (tPTX), and tPTX with autologous transplantation (tPTX + AT). All three procedures had clear and definite efficacy in reducing PTH levels and improving patients’ clinical symptoms [7, 8], with each having its own advantages and disadvantages. No prospective randomized study has compared the advantages and disadvantages of these three surgical procedures, and the choice of procedure depends on the surgeon’s experience. Among the three surgical procedure, the recurrence rate of sPTX and tPTX + AT was much higher than tPTX [9, 10]. In patients who remained on dialysis for a prolonged period, we hypothesized that active vitamin D supplementation could treat hypocalcemia caused by postoperative hypoparathyroidism. So, to minimize the risk for recurrence as well as the number of surgical interventions, we perform tPTX without autotransplantation in patients who were ineligible for renal transplantation. From January 2018 onwards, the SHPT-MDT team of Yuxi People’s Hospital, with the Department of Nephrology and the Department of Otolaryngology-Head and Neck Surgery as the core, has performed total parathyroidectomy (tPTX) in 34 patients with refractory SHPT and obtained favorable clinical outcomes, which are reported below.

### Data and methodology

#### Patient selection

In the past year, our team has performed 41 surgical resections of the parathyroid glands. We followed 34 consecutive patients who had undergone tPTX without autotransplantation for SHPT in the Department of Nephrology, Yuxi People’s Hospital, Yunnan Province, China, from January 2018 to October 2020. Twenty-five patients received hemodialysis, and nine patients received peritoneal dialysis at the time of tPTX. No patient had a functioning renal transplant at the time of tPTX. For seven other patients, tPTX was not intended (i.e., tPTX with autotransplantation); thus, they were excluded from the analyses.

#### Operative technique and sampling intraoperative PTH

**Fig. 1:**
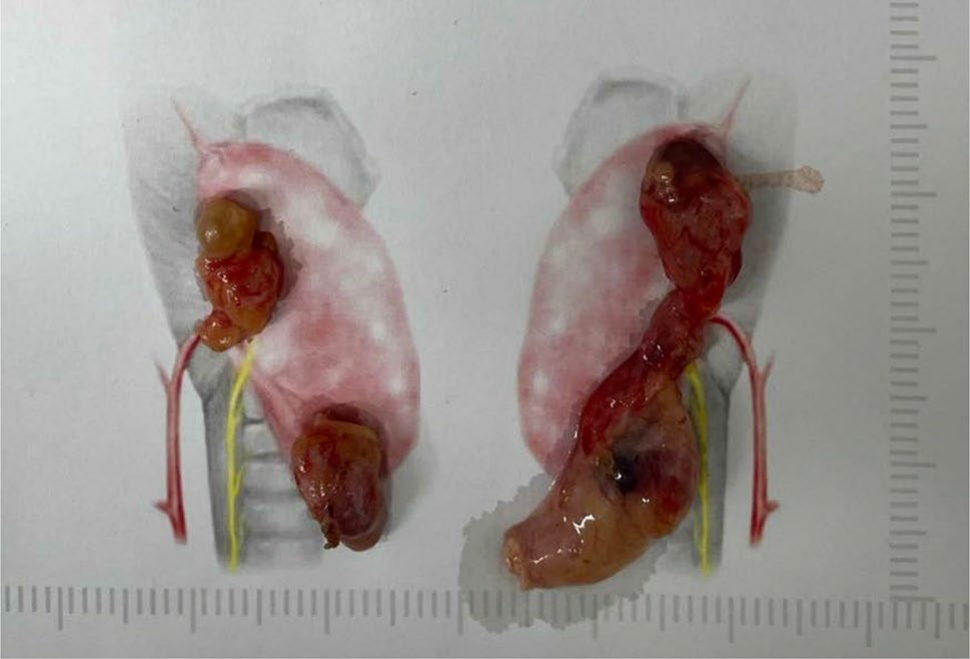
Parathyroid glands resected after bilateral neck exploration

Standard bilateral neck exploration [11] was adopted for bilateral parathyroid exploration and resection. The specific procedures were as follows: the thyroid gland was exposed to thyroid surgery, and the thyroid lobes were retracted medially and finely dissected along the fibrous capsule of the thyroid gland using a bipolar electrocoagulator. A meticulous search was performed for the parathyroid glands in the following order: normal anatomic region, expanded normal region, acquired migrated region, and congenital ectopic region of the inferior and superior parathyroid glands. If four or more parathyroid glands were found, the procedure was terminated (Fig. 1). Thymectomy and exploration of the carotid sheath were performed only in cases with less than four identified glands. During tPTX, samples were drawn 0, 10, 20, and 40 min after removal of all specimens. Intraoperative PTH was measured immediately by the Laboratory of Nuclear Medicine (iPTH, chemiluminescent immunoassay method; DiaSorin LIASON, Italy).

### Data collection

Data were obtained from the patients’ charts and computer-based records: (1) demographic details, including sex, age, underlying renal disease, and indication for tPTX; (2) date of diagnosis of chronic renal failure, start or change of dialysis treatment, tPTX, renal transplant, or last follow-up; (3) preoperative details, including clinical features such as bone pain and visible extraskeletal calcifications; diagnostic measures such as skeletal radiology and bone mineral density; biochemistry including calcium, phosphate, and iPTH levels; and (4) information regarding the PTX procedure, including the number of glands identified and removed and histological appearance of the removed tissue, and (5) changes in major symptoms, complications, and serial biochemistry postoperatively.

### Statistical analysis

Statistical analysis was performed using SPSS 22.0. Analysis of variance was used for intergroup comparisons of the normally distributed measures. The Friedman test was used to compare the preoperative and postoperative changes in iPTH, calcium, and phosphorus levels. *P* < 0.05 was considered statistically significant.

## Results

### Patients and indications for tPTX

From January 2018 to October 2020, 34 patients (22 men and 12 women) underwent tPTX without autotransplantation, were aged 29–65 (45.56 ± 11.14, $\mathrm{mean}\pm \mathrm{SD}$) years, and had dialysis vintage of 16–151 (86.55 ± 32.16, $\mathrm{mean}\pm \mathrm{SD}$) months. The underlying renal disease was chronic glomerulonephritis in 23 cases, hypertensive renal damage in eight cases, diabetic nephropathy in one case, gouty nephropathy in one case, and congenital solitary kidney in one case.

The indications for tPTX were hyperparathyroid bone disease in all cases, with skeletal deformities observed in eight cases (23.63%), “shrinking man” syndrome observed in three cases (8.82%), which manifested as a shortening of height by > 10 cm, and a history of multiple fractures noted in three cases (8.82%). The preoperative iPTH level was 1657.38 ± 871.33($\mathrm{mean}\pm \mathrm{SD}$) pg/mL (range, 757.61–26,100.00 pg/mL) (Table 1).

**Table 1:** Baseline characteristics of patients and biochemical analyses

Parameter	*N* = 34
Age (y,$\mathrm{mean}\pm \mathrm{SD}$)	45.56 ± 11.14
Male (*N*, %)	22 (64.70)
CKD etiology (*N*, %)	
Glomerulonephritis	23 (67.65)
Hypertension	8 (23.53)
Diabetes mellitus	1 (2.94)
Gouty nephropathy	1 (2.94)
Congenital single kidney	1 (2.94)
Dialysis vintage (months,$\mathrm{mean}\pm \mathrm{SD}$)	86.6 ± 32.2
Type of RRT (*N*, %)	
Hemodialysis	25 (73.53)
Peritoneal dialysis	9 (26.47)
Ionic Ca (mmol/L,$\mathrm{mean}\pm \mathrm{SD}$)	1.17 ± 0.08
Total Ca (mmol/L,$\mathrm{mean}\pm \mathrm{SD}$)	2.33 ± 0.18
Phosphate (mmol/L,$\mathrm{mean}\pm \mathrm{SD}$)	2.15 ± 0.57
Ca*P (mg/dL,$\mathrm{mean}\pm \mathrm{SD}$)	62.27 ± 17.39
Pre-iPTH (pg/mL, $\mathrm{mean}\pm \mathrm{SD}$)	1657.38 ± 871.33
Alkaline phosphatase (IU/L,$\mathrm{mean}\pm \mathrm{SD}$)	481.21 ± 520.33
Hemoglobin (g/L,$\mathrm{mean}\pm \mathrm{SD}$)	104.63 ± 23.81
Serum albumin (g/L, $\mathrm{mean}\pm \mathrm{SD}$)	40.22 ± 5.53

*N* number; *CKD* chronic kidney disease; *iPTH* intact parathyroid hormone; *Ca* calcium; Ca*P calcium–phosphorus product

### Number and distribution of parathyroid glands

In 34 patients, 142 parathyroid glands were removed, which were confirmed to be hyperplastic or even adenomatous via postoperative pathology. There were 121 (85.21%) parathyroid glands located in the bilateral tracheoesophageal groove and 21 (14.79%) ectopic parathyroid glands. Ten ectopic parathyroid glands were located in the lingual lobes of the thymus, five in the thymus, three in the thyroid, one in the carotid sheath, one in the parapharyngeal space, and one posterior to the esophagus (Table 2). Six parathyroid glands were removed in one case (2.94%), five glands were removed in five (14.70%), four were removed in 27 (79.41%), and three were removed in one (2.94%). Moreover, 17.64% (6/34) of the patients had supernumerary parathyroid glands.

**Table 2:** Number and distribution of parathyroid glands

Location of the parathyroid glands	Nmber (*n* = 142)
Tracheoesophageal groove (*N*, %)	121 (85.21)
Ectopic parathyroid glands (*N*, %)	21 (14.79)
Lingual lobe of thymus (*N*, %)	10 (7.04)
Thymus (*N*, %)	5 (3.52)
Thyroid (*N*, %)	3 (2.11)
Carotid sheath (*N*, %)	1 (0.70)
Parapharyngeal space (*N*, %)	1 (0.70)
Posterior to esophageal (*N*, %)	1 (0.70)

### Postoperative course, persistency, and recurrence

At 6 h postoperatively, bone pain and arthralgia were significantly alleviated and almost disappeared in 94.12% (32/34) of patients. At 24 h postoperatively, bone pain was relieved, limb movement was improved in 100% (34/34) of patients, and pruritus almost disappeared in 86.36% (19/22) of patients. Within 1 week postoperatively, patients with restless leg syndrome showed a significant decrease in the frequency and intensity of episodes, and patients with muscle weakness and insomnia had obvious symptom improvement. Bone pain, arthralgia, and pruritus in all patients disappeared at 6 months postoperatively, with significant improvement in muscle weakness, sleep quality, and restless leg symptoms.

**Fig. 2:**
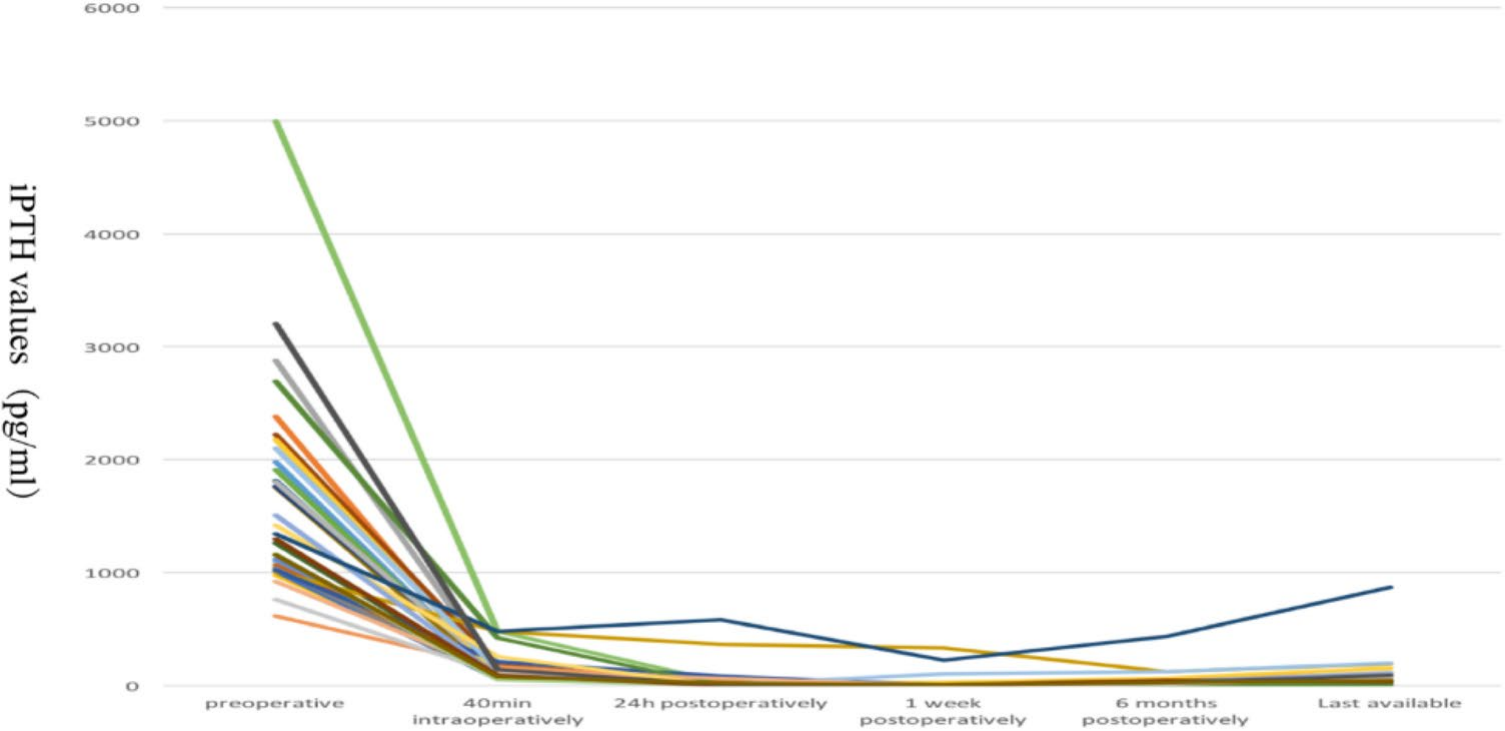
Pre- and postoperative iPTH levels in 34 patients after tPTX

Postoperative iPTH levels were available in all patients (Fig. 2). The Friedman test was used to analyze the effect of surgery on iPTH, calcium, and phosphorus levels. The results revealed significant differences in iPTH (*χ*2 = 134.93, *P* < 0.05), calcium (*χ*2 = 23.02, *P* < 0.05), and phosphorus (*χ*2 = 102.11, *P* < 0.05) levels preoperatively, 40 min intraoperatively, and 24 h, 1 week, 6 months, and last available (> 1 year) postoperatively (Table 3). Pairwise comparative analysis suggested statistically significant differences in iPTH levels between patients preoperatively, 40 min intraoperatively, 24 h postoperatively, 1 week postoperatively, 6 months postoperatively, and last available, postoperatively (*P* < 0.05). There were statistically significant differences in calcium levels preoperatively, 24 h postoperatively, 1 week postoperatively, 6 months postoperatively, and last available postoperatively (*P* < 0.05). There were statistically significant differences in phosphorus levels preoperatively, 40 min intraoperatively, 1 week postoperatively (median, 1.04 mmol/L), 6 months postoperatively, and last available postoperatively (*P* < 0.05).

**Table 3:** Changes in iPTH, blood calcium, and blood phosphorus levels before and after PTX

	Preoperative	40 min intraoperatively	24 h postoperatively	1 week postoperatively	6 months postoperatively	Last available	*P*-value
iPTH (pg/mL) median(p25, p75)	1338.10 (1054.30, 2038.70)	106.20 (71.85, 141.55)	9.68 (2.66, 35.12)	1.47 (1.00, 8.77)	17.88 (8.06, 35.45)	43.32 (26.11, 88.73)	0.00
Calcium mmol/L,) median(p25,p75)	2.36 (2.26, 2.44)	2.24 (1.88, 2.52)	2.04 (1.89, 2.20)	2.10 (1.98, 2.28)	2.05 (1.95, 2.28)	2.03 (1.86, 2.34)	0.00
Phosphorus (mmol/L) median(p25,p75)	2.32 (1.64, 2.66)	1.67 (1.28, 1.94)	1.64 (1.22, 1.98)	1.04 (0.88, 1.20)	1.11 (0.96, 1.44)	1.29 (1.01, 1.46)	0.00

One patient had four hyperplastic parathyroid glands removed after bilateral neck exploration; however, the postoperative PTH level did not decrease to < 60.00 pg/mL and increased to 870.78 pg/mL 1 year, postoperatively. The patient had persistent hyperparathyroidism. In other patients, PTH levels decreased to < 60.00 pg/mL 24 h postoperatively and did not increase to > 150.00 pg/mL during the follow-up period.

### Postoperative complications and follow-up

One case revealed postoperative hypercalcemia, and the blood calcium level decreased to normal 48 h postoperatively after adjustment for calcium supplementation. Eleven cases experienced postoperative hypocalcemia (32.35%, 11/34), including four of severe hypocalcemia (11.76%, 4/34) and no permanent hypocalcemia. No patient with hypocalcemia developed severe symptoms, such as hypocalcemic convulsions, spasms, or arrhythmias. Patients with hypocalcemia had a smooth transition to normal blood calcium levels after intravenous supplementation with 10% calcium gluconate and oral administration of calcium supplements and active vitamin D. Transient hoarseness was observed in one case, which recovered 1 month postoperatively without permanent vocal cord paralysis, laryngeal obstruction, choking on water, bleeding, wound effusion, or infection. No patient had acute myocardial infarction or deep vein thrombosis.

After a median follow-up of 33 (range, 15–47) months, hypoparathyroidism was observed in three cases, all of which exhibited improvements after vitamin D supplementation, with no hypocalcemia or adynamic bone disease. No deaths occurred during the follow-up period.

## Discussion

Surgical treatment is playing a more and more important role in SHPT. For refractory SHPT, PTX is recommended in most practical guidelines, but the best available surgical option for SHPT is still greatly debated. Postoperative hypocalcemia (or hypoparathyroidism) and recurrence are always unavoidable in the surgical treatment of SHPT. Although it entails some fear of adynamic bone disease in cases of hypoparathyroidism, as well as an unwillingness to constantly supplement patients with active vitamin D postoperatively, tPTX without autotransplantation has gained increasing popularity in recent years [12–14]. In our study, after tPTX was implemented, 31/34 patients (91.18%) did not develop hypoparathyroidism and did not need calcium and vitamin D supplements. Only 3/34 (8.82%) patients with iPTH levels below the normal range, calcium and phosphorus metabolism was improved by calcium and vitamin D treatment, and no dynamic bone disease occurred. Similarly, as described by others [8, 15, 16], compared with sPTX and tPTX + AT, tPTX is proven to be effective and safe, but has obvious advantages in reducing the recurrence.

Another concern with tPTX stems from patients receiving postoperative kidney transplants. In a study by Puccini et al. [12], no permanent hypoparathyroidism was observed in patients who received kidney transplants after tPTX. A long-term follow-up study by Rayes et al. [17] also confirmed that kidney transplant recipients benefited from tPTX. Meanwhile, many studies have shown that excessive PTH levels, both before and after kidney transplantation, are detrimental to the survival and functioning of the transplanted kidney. No patients underwent kidney transplantation after tPTX, nor did the PTH levels decrease again due to other postoperative treatments or medications. However, some studies have shown that PTH levels can decrease again after kidney transplantation and that PTH can even decrease to the lower limit of the normal range after kidney transplantation in some patients, which requires long-term maintenance treatment via oral administration of active vitamin D and calcium [18]. Therefore, it is important to consider whether a patient has the prospect of kidney transplantation when making surgical decisions. Further studies are needed to determine the safety of tPTX in patients undergoing kidney transplantation.

The 34 patients in this study had significant symptomatic relief on the first postoperative day. Patients were followed-up for 15–47 (median 33) months. PTH, serum calcium, and phosphorus levels were monitored, and all were stable. No cases of adynamic bone disease or deaths were observed. Numerous studies worldwide [8, 19, 20] have also confirmed the efficacy of tPTX in the treatment of SHPT, which had a low recurrence rate and is safe and reliable, with no postoperative complications such as hypocalcemia, hypoparathyroidism, and adynamic bone disease. Therefore, tPTX may be the procedure of choice for patients with renal SHPT who are ineligible for or have no hope of renal transplantation.

Regardless of the procedure, the primary goal of surgical treatment for SHPT is to reduce the PTH levels to eliminate clinical symptoms and halt the target organ damage. All three procedures unquestionably reduced PTH levels. However, to what level, at what level, and for how long can PTH be reduced and maintained? Major differences between the procedures remain. In this study, except for one case of persistent hyperparathyroidism and three of low postoperative PTH levels, all patients maintained a desirable postoperative PTH levels, and all clinical symptoms improved postoperatively, with no adynamic bone disease or death identified during the follow-up. The Kidney Disease: Improving Global Outcomes (KDIGO) guidelines and the Guidance for Diagnosis and Treatment of Chronic Kidney Disease-Mineral and Bone Disorder in China have set the PTH target values for patients on maintenance dialysis at two to nine times the upper limit of the normal range [21, 22], without further recommendations on the PTH target levels specifically for post-PTX patients on maintenance dialysis. Were such target values based on evidence-based studies to improve the quality of life and extend the survival of patients or did technical limitations prevent maintaining PTH at normal levels or slightly above the normal? Iwamoto et al. [23] found that patients in a post-PTX iPTH < 16.6 pg/mL group had a significantly higher overall survival rate than those in a ≥ 16.6 pg/mL group. A study by Zhang et al. [24]also suggested that all-cause mortality was lowest when postoperative iPTH levels were controlled between 21 and 150 pg/mL. The recommended iPTH levels for dialysis patients in the current guidelines are unsuitable for post-PTX patients and it is necessary to determine the control range of iPTH levels and the ideal calcium and phosphorus metabolism indicators in post-PTX patients via more rigorously designed studies with longer follow-up periods.

In this study, three patients had low postoperative PTH levels. Although no symptoms of hypoparathyroidism were observed and the blood calcium and other biochemical indicators remained within their respective ideal levels, the low postoperative PTH levels were detrimental to the long-term survival of the patients [24]. Among these three patients, partial thymotomy was performed simultaneously as supernumerary or ectopic glands were found in two patients, and one underwent central dissection for combined thyroid cancer. The inferior parathyroid gland migrates downward with the thymus during embryonic development and forms some parathyroid cell remnants, approximately 37% of which are located in the thymus [25]. Therefore, to prevent recurrence, exploration of the thymus is essential during PTX to locate and remove most ectopic or supernumerary parathyroid glands. Despite this, the normal thymus tissue must be properly preserved to prevent permanent hypoparathyroidism as it is a potential source of PTH after tPTX.

All 34 patients in this group received tPTX, and one case with postoperative persistent hyperparathyroidism was observed. Although only three parathyroid glands were localized preoperatively in this patient, four hyperplastic parathyroid glands were removed via standard bilateral neck exploration. Intraoperative PTH (ioPTH) monitoring suggested that the ioPTH 10 min (502.80 pg/mL), ioPTH 20 min (480.53 pg/mL), and ioPTH 40 min (478.21 pg/mL) after the excision of the last PT observed in exploration were reduced by approximately 70% from the preoperative baseline values (1388.32 pg/mL). However, the decrease in ioPTH 20 min and ioPTH 40 min compared with ioPTH 10 min was insignificant, and both remained at high levels. Therefore, it is not advisable to simply copy the Vienna or Miami criteria [26] to determine whether the hyperfunctional glands are cleared in SHPT. Moreover, the metabolic characteristics of SHPT patients may affect the half-life of iPTH [27], making it inappropriate to adopt the Halle or Rome criteria, which are more suitable for multiglandular lesions [26]. The ioPTH monitoring evaluation of SHPT should combine the decreased level of ioPTH at 10 min from the baseline and the decreasing curve between ioPTH at 20 min and ioPTH at 40 min to determine whether the hyperfunctional glands have been completely removed. Combining the data of ioPTH monitoring and the follow-up results of the present study, an ioPTH 10 min decrease of > 70% from the baseline value and persistent decrease in ioPTH 20 min and ioPTH 40 min with a level < 20% of the baseline value should be reliable in determining whether the hyperfunctional glands have been removed.

Limitations: first, this study was a retrospective study with a relatively small sample size; therefore, our results require verification through further clinical studies with larger populations. Second, this was not a double-blinded randomized study; thus, selection biases might have occurred. Further prospective studies are required to address these issues. A longer follow-up period will also help determine the effectiveness of tPTX in the long-term control of hyperparathyroidism. Finally, the subjects included were only Chinese, which limited the conclusions applicable to other ethnic groups.

In conclusion, tPTX was effective in the treatment of SHPT with a low recurrence rate, good safety and reliability, and no postoperative complications, such as intractable hypocalcemia, permanent hypoparathyroidism, and adynamic bone disease. Thus, tPTX may be the procedure of choice for patients with renal SHPT who are not eligible for kidney transplantation.
